# Study protocol of a multiphase optimization strategy trial (MOST) for delivery of smoking cessation treatment in lung cancer screening settings

**DOI:** 10.1186/s13063-022-06568-3

**Published:** 2022-08-17

**Authors:** Jamie S. Ostroff, Donna R. Shelley, Lou-Anne Chichester, Jennifer C. King, Yuelin Li, Elizabeth Schofield, Andrew Ciupek, Angela Criswell, Rashmi Acharya, Smita C. Banerjee, Elena B. Elkin, Kathleen Lynch, Bryan J. Weiner, Irene Orlow, Chloé M. Martin, Sharon V. Chan, Victoria Frederico, Phillip Camille, Susan Holland, Jessica Kenney

**Affiliations:** 1 Department of Psychiatry and Behavioral Sciences, Memorial Sloan Kettering Cancer Center, 641 Lexington Ave., 7th Floor, New York, NY 10022 USA; 2 School of Global Public Health, New York University, New York, USA; 3 GO2 Foundation for Lung Cancer, Washington, D.C., USA; 4 Department of Health Policy and Management, Columbia Mailman School of Public Health, New York, USA; 5 Department of Global Health, University of Washington, Seattle, USA

**Keywords:** Smoking cessation, Tobacco treatment, Lung cancer screening, Pragmatic clinical trial, Implementation science

## Abstract

**Background:**

There is widespread agreement that the integration of cessation services in lung cancer screening (LCS) is essential for achieving the full benefits of LCS with low-dose computed tomography (LDCT). There is a formidable knowledge gap about how to best design feasible, effective, and scalable cessation services in LCS facilities. A collective of NCI-funded clinical trials addressing this gap is the Smoking Cessation at Lung Examination (SCALE) Collaboration.

**Methods:**

The Cessation and Screening to Save Lives (CASTL) trial seeks to advance knowledge about the reach, effectiveness, and implementation of tobacco treatment in lung cancer screening. We describe the rationale, design, evaluation plan, and interventions tested in this multiphase optimization strategy trial (MOST). A total of 1152 screening-eligible current smokers are being recruited from 18 LCS sites (*n* = 64/site) in both academic and community settings across the USA. Participants receive enhanced standard care (cessation advice and referral to the national Quitline) and are randomized to receive additional tobacco treatment components (motivational counseling, nicotine replacement patches/lozenges, message framing). The primary outcome is biochemically validated, abstinence at 6 months follow-up. Secondary outcomes are self-reported smoking abstinence, quit attempts, and smoking reduction at 3 and 6 months. Guided by the Implementation Outcomes Framework (IOF), our evaluation includes measurement of implementation processes (reach, fidelity, acceptability, appropriateness, sustainability, and cost).

**Conclusion:**

We will identify effective treatment components for delivery by LCS sites. The findings will guide the assembly of an optimized smoking cessation package that achieves superior cessation outcomes. Future trials can examine the strategies for wider implementation of tobacco treatment in LDCT-LCS sites.

**Trial registration:**

ClinicalTrials.gov NCT03315910

## Administrative information

**Table:** 

**Title**	Study protocol of a multiphase optimization strategy trial for delivery of smoking cessation treatment in lung cancer screening settings
**Trial Registration**	Clinicaltrials.gov: NCT03315910
**Protocol version**	A(13) Approved 03/03/2022
**Funding**	This work was supported by grants from the National Cancer Institute (NCI). The clinical trial was supported by an R01 grant (R01CA207442) awarded to Drs. Ostroff and Shelley (MPIs) in response to RFA #15-011 Smoking Cessation within the Context of Lung Cancer Screening. All eight funded projects formed the SCALE (Smoking Cessation at Lung Examination) Collaboration being coordinated by the NCI. A Diversity Supplement supported an ancillary study focusing on identifying challenges to the recruitment of Black smokers. The work was also supported by the PRO-CEL Core Facility supported by MSK’s Cancer Center Support Grant (P30CA008748).
**Author details**	Jamie S. Ostroff, PhD^1^, Donna Shelley, MPH, MD^2^, Lou-Anne Chichester^1^, Jennifer C. King^3^, Yuelin Li^1^, Elizabeth Schofield^1^, Andrew Ciupek^3^, Angela Criswell^3^, Rashmi Acharya^3^, Smita C. Banerjee^1^, Elena Elkin^4^, Kathleen Lynch^1^, Bryan J. Weiner^5^, Irene Orlow^1^, Chloé M. Martin^1^, Sharon Chan^1^, Victoria Frederico^1^, Phillip Camille^1^, Susan Holland^1^, Jessica Kenney^1^ Department of Psychiatry and Behavioral Sciences, Memorial Sloan Kettering Cancer Center^1^; School of Global Public Health, New York University^2^; GO2 Foundation for Lung Cancer^3^; Department of Health Policy and Management, Columbia Mailman School of Public Health^4^; Department of Global Health, University of Washington^5^
**Name and contact information for trial sponsor**	Memorial Sloan Kettering Cancer Center (MSK) 1275 York Ave, New York, NY 10065
**Role of sponsor**	The study sponsor, MSK, serves as the data coordinating center for this study. In addition to working with collaborators to develop the study, the MSK research team handles the day-to-day management of the study, including participant consenting and randomization, data collection, management, and analysis. MSK will also organize and lead team meetings designed to provide study oversight and will be responsible for all data sharing and dissemination of study findings.

## Introduction

In response to the landmark National Lung Screening Trial [1], the United States Preventive Services Task Force [2] (USPSTF) recommends annual low-dose computed tomography lung cancer screening (LDCT-LCS) for high-risk individuals (adults 55–80 years of age with 30 pack per year smoking history who currently smoke or have quit within the past 15 years). These guidelines were revised in 2021 such that adults aged 50–80 years with a 20-pack-year smoking history who currently smoke or quit within the past 15 years are now recommended for LDCT-LCS [3]. There are an estimated 14.5 million Americans eligible for screening and approximately 50% are likely people who currently smoke [4].

LDCT-LCS provides an unprecedented opportunity to further reduce tobacco-related morbidity and mortality by delivering smoking cessation treatment within the context of lung cancer screening [5, 6]. There is strong support for the integration of tobacco treatment as an indicator of high-quality LCS [7] and the cost-effectiveness of LDCT-LCS is enhanced when paired with tobacco cessation counseling [8, 9].

Although clinical practice guidelines for the treatment of tobacco use and dependence exist [10], access to and utilization of tobacco treatment services within the context of LCS has been highly variable [11]. Little is known about the readiness and capacity of LCS sites to deliver evidence-based tobacco treatment. There is also a knowledge gap in identifying the reach, effectiveness, implementation and sustainability of various treatment approaches [12]. Studies that have examined the effectiveness of providing only brief cessation advice and/or brief cessation interventions (i.e., brochure, brief counseling, Quitline referral) have generally been under-powered and failed to produce significant findings [13–15]. For LDCT-LCS sites to integrate tobacco treatment into their screening protocols, it is also critical to understand implementation costs and workflows [6]. Although a simulation-based cost-utility analysis strongly supports the value of smoking cessation [16], the incremental costs of delivering treatment within LDCT-LCS are largely unknown. Finally, in order to mitigate tobacco-related disparities, it is essential to reach and engage people who smoke from diverse racial and ethnic backgrounds in tobacco treatment [17]. Recruiting diverse populations of people with smoking histories in tobacco cessation trials is often challenging, particularly for the engagement of Black people who smoke [18].

Eight (8) projects were awarded National Cancer Institute (NCI) grants in response to RFA #15-011, and the teams of these projects have formed the SCALE Collaboration, coordinated by the NCI [19]. As one of the SCALE studies, the Cessation and Screening to Save Lives (CASTL) trial was designed to advance the science of tobacco treatment effectiveness and implementation in the LCS context. Here, we present the study design, tobacco treatment protocols, and evaluation plan of the CASTL trial, including an ancillary investigation focusing on the understanding of recruitment and engagement of Black people seeking LDCT-LCS in tobacco treatment.

## Methods

### Overview of CASTL study design

**Fig. 1:**
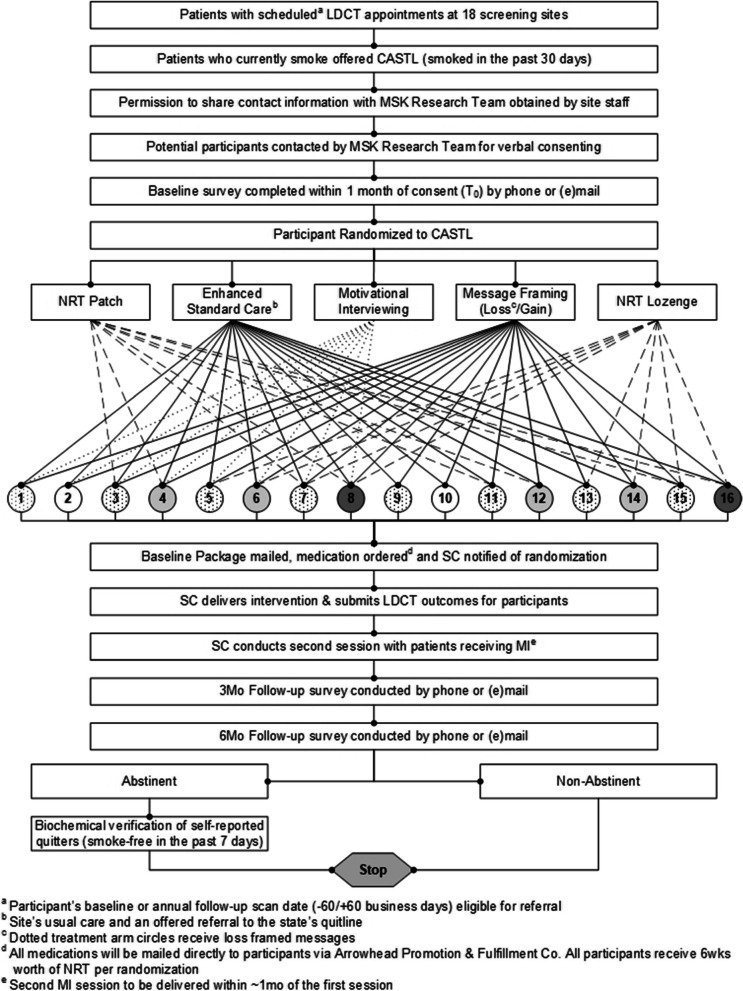
Study design

The CASTL Project utilizes a multiphase optimization strategy trial (MOST) [20] conducted at 18 lung cancer screening sites located across the USA. The MOST methodology leverages a full-factorial or a fractional-factorial design to systematically and efficiently evaluate the effects of individual treatment components and their interactions and then select those that produce the most favorable net effects [20–22]. The MOST enables participants to be randomized into one of 16 combinations of intervention components, stratified by participating site. The study design is depicted in Fig. 1. All participants receive enhanced standard care (i.e., cessation advice and referral to the national Quitline) and are randomly assigned to receive additional tobacco treatment components alone or in combination (i.e., motivational counseling, nicotine replacement patch, nicotine replacement lozenges, message framing booster). This factorial design enables the estimation of the main effect contribution of each of the four treatment components and interactions between components [23, 24]. Each screening site is managed by one or more site coordinators (SC). Each of the 18 participating sites is expected to recruit 64 patients who smoke and are eligible for lung cancer screening with LDCT (total target *N* = 1152). Surveys and qualitative interviews are being conducted with participating patients and staff. The primary outcome is biochemically validated, smoking abstinence at 6 months follow-up. Secondary cessation outcomes are self-reported smoking abstinence, quit attempts, and smoking reduction at 3- and 6-month follow-up. Guided by Proctor’s Implementation Outcomes Framework (IOF) [25], our evaluation plan includes measurement of implementation process outcomes including the treatment’s reach of the target population, fidelity, acceptability, appropriateness, sustainability, and cost.

### Study aims

#### Aim 1

To identify which of four evidence-based tobacco treatment components contribute to superior cessation endpoints among people who currently smoke and are seeking lung cancer screening over and above enhanced standard care (i.e., minimum intervention needed for change). The four tobacco treatment components being tested are (1) motivational interviewing (MI) (yes vs. no), (2) nicotine replacement therapy (NRT) patch (yes vs. no), (3) NRT lozenge (yes vs. no), and (4) message framing (gain vs. loss). We hypothesize that all four treatment component enhancements will be statistically superior to enhanced standard care alone and that some synergistic treatment component effects will emerge. For the message framing condition, we expect that gain-framed messages will result in higher quit rates. The primary cessation outcome is biochemically verified 7-day point abstinence at 6 months following study enrollment. Secondary cessation outcomes are self-reported 7-day point prevalence abstinence at 3 months, continuous abstinence (between the date of randomization and date of completion of follow-up surveys) at 3 and 6 months, and self-reported 24-h quit attempt at 3 and 6 months.

#### Aim 2

To estimate the cost and incremental cost-effectiveness of evidence-based tobacco treatment components, delivered alone and in combination.

#### Aim 3

To conduct a robust, mixed methods evaluation of the implementation process and assess site characteristics and organizational factors that may influence the implementation of tobacco treatment in lung cancer screening settings.

#### Aim 4

To analyze whether lung cancer screening results (i.e., Lung-RADS®) moderate smoking abstinence outcomes. We hypothesize that participants found to have one or more screening abnormalities will be more likely to quit smoking and maintain smoking abstinence.

#### Ancillary study—Participation of African-Americans in Cessation Trial (PACT)

To gain a better understanding of factors impacting trial refusal among Black individuals who smoke using a rigorous qualitative approach. This ancillary study was funded by an NCI Diversity Supplement.

### Conceptual framework

Two complementary evaluation frameworks, Proctor’s Implementation Outcomes Framework [25] and the Consolidated Framework for Implementation Research (CFIR), guide our assessment plan and selection of implementation outcomes [26]. Proctor’s Framework measures include (1) the intervention’s reach into the target population, (2) adoption (uptake of the intervention), (3) implementation fidelity, (4) acceptability (perception that treatment is satisfactory), (5) appropriateness (intervention fit/workflow compatibility), (6) sustainability (potential for sustained use/integration and barriers to achieving durability), and (7) cost [25]. We will also assess the organizational characteristics or “inner settings” constructs defined by the Consolidated Framework for Implementation Research (e.g., organizational priority, implementation climate, resources, leadership) that may influence the implementation of smoking cessation treatment in lung cancer settings [26].

### Study setting and site recruitment

This trial is being conducted at 17 LDCT-LCS sites across the USA. Recruitment of participating sites and site coordinators (SC) was led by the staff from the GO2 Foundation for Lung Cancer (GO2) (formerly Lung Cancer Alliance). Sites were eligible for participation in the CASTL trial if they were part of GO2’s Screening Centers of Excellence (SCOE) national network indicating having met several criteria for high-quality screening practices. Study sites were required to have at least 1 year of lung cancer screening experience, employ at least one SC, and report conducting at least 20 LDCT screenings per month so as to have an adequate patient volume for achieving the target recruitment of patients who smoke during the allotted study time frame. To promote participant diversity, there was attention paid to selecting a wide variety of LDCT-LCS sites both with and without academic affiliations and with geographic diversity across the USA. LDCT-LCS sites were recruited and onboarded in staggered waves.

### Patient eligibility

Consistent with the 2013 USPSTF recommendations for LDCT-LCS, eligible patient participants are between [27] the ages of 55–80 years old (at the time of their LDCT scan), seeking baseline or annual follow-up LDCT-LCS, have at least a 30-pack-year history of smoking, currently smoking (defined as self-reported cigarette smoking on some days or every day) within the past 30 days, reachable by telephone, and English speaking. In April 2021, the eligibility criteria were expanded to include patients who were between the ages of 50–80 years old (at the time of their scan), with at least a 20-pack-year history of smoking. To facilitate the inclusion of non-English speaking participants, the study materials were later translated into Spanish and Spanish-English bilingual staff were hired. Patients are excluded from participation if either nicotine replacement therapy (NRT) is medically contraindicated (e.g., recent heart attack within the last 2 weeks) or they presented severe medical or psychiatric comorbidities that may prevent them from participating fully in the study (per the discretion of the screening SC or the study’s principal investigators). Finally, patients who are receiving concurrent tobacco treatment services or have used cessation medications (NRT, bupropion, varenicline) within the past month with the intent to quit smoking are excluded. Black people who smoke who actively or passively refused participation in CASTL are approached for participation in the ancillary refusal study.

### Participant recruitment

Each site has a target enrollment of 64 participants (total *n* = 1152). All participants who report current smoking are identified by the SCs (or designee) who introduce the CASTL study to all eligible patients during scheduling for their LDCT scan. SCs provide names and contact information of interested patients to the Memorial Sloan Kettering Cancer Center (MSK) study staff daily. SCs also maintain a detailed screening log of all people reporting current smoking with LDCT-LCS appointments and provide weekly de-identified updates regarding the number of people currently smoking who were ineligible for CASTL and the reasons for ineligibility. Information reported includes the number of refusers, their reasons for refusal, and limited demographic characteristics (age, gender, race, and ethnicity) of all people who currently smoke seeking LDCT-LCS. These screening logs provide the information needed to determine the reach rate, the proportion of eligible, participants who enroll in the CASTL trial.

An MSK Clinical Research Coordinator (CRC) emails potential participants a study introduction letter shortly after interested participants are referred by the SCs and makes up to 3 telephone attempts to confirm their eligibility. Trained CRCs have a non-written (verbal) informed consent discussion with participants so as to confirm eligibility prior to randomization. Participants will receive study information by mail following these consent discussions. Initially, consent was obtained prior to their lung cancer screening visit, or within seven business days of the completion of their LDCT scan. However, the COVID-19 pandemic issues resulted in LDCT-LCS sites reporting closures, scheduling delays, and disruptions requiring a protocol amendment to allow for enrollment of eligible patients anytime in LDCT clinical workflow between 60 business days prior to the LDCT appointment and 60 business days following the scan.

Eligible Black patients who actively or passively refuse CASTL participation (patients who do not give permission to be contacted by the MSK coordinating staff) are given a brochure containing descriptive information about the ancillary refuser study and encouraged to contact the MSK study team by email or telephone. In addition, Black patients who initially gave permission to be contacted but subsequently actively or passively refuse participation in the CASTL trial are provided with a verbal description of the PACT study either during the telephone contact or emailed a description of the PACT study once all CASTL recruitment attempts have been made in an effort to engage them in the ancillary study. PACT participants will be verbally consented by the research scholar leading this ancillary study.

### Patient randomization and assignment to treatment group

Eligible patients who smoke are registered via MSK’s Clinical Trials Management System to the CASTL trial and randomized using the Clinical Research Database by a CRC. Individual study participants are randomized to one of the 16 intervention combinations listed in Table 1. Randomization is stratified using the method of the random permuted block by participating LDCT-LCS site. After patient registration and randomization, a designated MSK CRC sends each SC a secure email containing the patient’s treatment assignment.

**Table 1:** Treatment components

Components					
Conditions	Enhanced standard care	Motivational interviewing	NRT lozenge	NRT patch	Message framing
**1**	Yes	Yes	No	No	Loss
**2**	Yes	Yes	No	No	Gain
**3**	Yes	Yes	No	Yes	Loss
**4**	Yes	Yes	No	Yes	Gain
**5**	Yes	Yes	Yes	No	Loss
**6**	Yes	Yes	Yes	No	Gain
**7**	Yes	Yes	Yes	Yes	Loss
**8**	Yes	Yes	Yes	Yes	Gain
**9**	Yes	No	No	No	Loss
**10**	Yes	No	No	No	Gain
**11**	Yes	No	No	Yes	Loss
**12**	Yes	No	No	Yes	Gain
**13**	Yes	No	Yes	No	Loss
**14**	Yes	No	Yes	No	Gain
**15**	Yes	No	Yes	Yes	Loss
**16**	Yes	No	Yes	Yes	Gain

#### Tobacco treatment components

Careful attention was given to the selection of tobacco treatment components guided by the following principles: (a) evidence-based, (b) feasible and scalable for high and low resource LDCT-LCS sites, and (c) sustainable beyond the CASTL study period. The study is designed to compare enhanced standard care (control group) with four tobacco treatment components: (1) motivational interviewing (MI) (yes vs. no), (2) nicotine replacement therapy (NRT) patch (yes vs. no), (3) NRT lozenge (yes vs. no), and (4) message framing (gain vs. loss).

##### Enhanced standard care

SCs provide all CASTL participants with self-help, print cessation materials targeting persons who currently smoke seeking lung cancer screening, *Why Quit Now? A Resource for Those at Risk for Lung Cancer* [28] and refer patients to the national Quitline at 1-800-QUIT NOW for education and follow-up cessation counseling during their first session within 1 month of randomization or during the shared decision-making discussion. Enhanced standard care was chosen as the comparator to ensure that at minimum, all participants receive cessation advice and an active referral to the Quitline, a service that was not consistently provided to participants prior to CASTL.

##### Motivational interviewing (MI) [29]

There is substantial evidence that even brief MI can increase adherence to tobacco treatment and quit rates [30]. Participants assigned to the MI component receive two MI counseling sessions delivered by the SC; the first delivered face to face or via telephone during the patient’s lung cancer screening visit, and the second session, delivered via telephone by the SC approximately 1 to 8 weeks after participants receive their LDCT scan results. In response to the difficulties reaching patients during the COVID-19 pandemic, we amended the protocol to allow SCs to deliver the first session of MI (including the enhanced standard care) within 1 month of the participant’s randomization to the study.

##### Nicotine lozenge [31, 32]

Participants randomized to this treatment component receive 6 cartons of 2 mg lozenges (81 units/carton) by mail, along with written instructions to use the lozenges to help manage acute nicotine withdrawal. Participants are instructed to use the NRT lozenges no more than every 1–2 h as needed and up to 20 lozenges per day during their initial session with the site coordinator for a period of 6 weeks.

##### Nicotine patch

Participants randomized to receive this treatment component receive 6 weeks of nicotine patch with dosing dependent upon baseline cigarettes per day, along with written instructions for safe and effective use by mail [31]. Participants who smoke fewer than 10 cigarettes per day receive 4 weeks of the 14 mg patch (2 boxes) and 2 weeks of the 7-mg patch (1 box). Those who smoke 10 or more cigarettes per day receive 2 weeks of the 21-mg patch (1 box), 2 weeks of the 14-mg patch (1 box), and 2 weeks of the 7-mg patch (1 box) and are instruction about proper usage during their initial session.

##### Message framing

Prior research has demonstrated that gain-framed messages (i.e., emphasize the benefits of quitting) may be more effective than loss-framed (i.e., emphasize the risks of persistent smoking) or non-framed (neutral) messages for encouraging smoking cessation [33, 34]. Within 1 month of receiving their LDCT-LCS results, participants receive a standardized message that emphasizes either the benefits of quitting (gain-framed) or the risks of continuing to smoke (loss-framed). Audio and video messages are pre-recorded by SCs and sent by email. For patients who do not have an email address, messages are sent via letter. A manipulation check assessing participant comprehension of the message framing intervention component is conducted.

### Training and treatment fidelity

To enhance the real-world generalizability of the trial findings, a trained SC from each LDCT-LCS site delivers the tobacco treatments. Several approaches are being used to enhance and measure fidelity to implementing enhanced standard care and the four treatment components following recommendations of the Treatment Fidelity Workgroup of the National Institute of Health Behavior Change Consortium [35, 36].

#### Initial training of LDCT-LCS SCs

All participating SCs receive 2.5-h didactic training in tobacco treatment via standardized webinar presentations led by one of the principal investigators and the research project associate. The webinar presentation covers the overall rationale for the integration of evidence-based smoking cessation in the context of lung cancer screening and specific training on the standard care provided for all CASTL participants as well as each of the 4 tobacco treatment enhancements being tested in the CASTL trial. The training includes information on the rationale for and proper use of NRT (lozenge and patch for the management of nicotine cravings and other symptoms of nicotine withdrawal). The training includes didactic training in brief MI and a demonstration video produced for the CASTL trial. SCs also receive an MI cessation counseling training manual that provides a detailed outline of recommended MI counseling sessions and are trained to complete an MI self-rating tool for each MI session conducted [37]. Lastly, each SC receives consultation regarding the development of an acceptable workflow individualized for routine LDCT-LCS referral to the quitline

During the CASTL trial, all participating SCs are invited to join twice monthly videoconference calls that cover presentation updates on the CASTL trial and SCALE collaboration, site recruitment, and pertinent reminders about protocol adherence and study implementation. The videoconference calls enable all participating SCs to have ongoing contact with the PIs and other members of the CASTL team and are intended to promote site retention and adherence to the study protocol.

#### Treatment implementation fidelity

The site coordinators document the delivery of the study interventions. Participants are also asked about the interventions they receive. To assess referrals to the Quitline, we obtain referral data from the SCs (e.g., fax referral forms). MSK research staff obtain a monthly utilization report from Arrowhead, the study-designated medication dispensary. This includes information such as which patient received NRT, when it was ordered, the exact package contents, and other usable data points. Study participants are also asked to keep a medication log to monitor NRT usage and use is assessed during the 3-month survey collection. SCs follow an MI treatment manual and provide self-assessments for MI adherence for those participants assigned to the MI condition [37]. Finally, the study staff document the distribution of the message framing letter and/or scripted video/audio.

### Data sources and timing of data collection

Data is collected from participating SCs and persons who currently smoke and are seeking lung cancer screening. Baseline SC surveys are completed prior to recruiting participants at their LCS sites. Following completion of target enrollment at each site, SCs complete a follow-up survey and a semi-structured interview. They also complete a semi-structured interview for the PACT ancillary study to assess recruitment practices during their active recruitment period for CASTL.

Baseline surveys are collected from eligible and consented participants prior to randomization. Two follow-up assessments are completed: the first occurring approximately 3 months and the second occurring approximately 6 months following randomization. Depending upon patient preference, surveys are being completed by phone (with an MSK research team member), by email (via REDCap), or by paper (by mail). Patient participants receive a $25 incentive for each assessment completed, including the return of the saliva sample for biochemical verification of smoking abstinence. Members of the MSK research team, including the data analysts are not blind to the treatment assignment. Data collection measures are listed in Table 2. To minimize attrition, participants receive a combination of up to six reminder phone calls, and/or emails, based upon their preference, for follow-up data collection. Participants who are unreachable after these attempts are mailed a hard copy of the survey for completion.

**Table 2:** Measures

Domains	Patient assessment				Site coordinator assessment		Instruments
	Baseline	3 months	6 months	Biochem	Baseline	Follow-up and interview	
Demographics							
Age, gender, race, ethnicity, education, marital status, SES, employment status^a^, income^a^	X				X		
Health literacy	X						BRIEF [38]
General health							HINTS [39] EQ 5D [40] Charlson Comorbidity Index [41]
Physical health	X						
Medical co-morbidities	X						
Family history of lung cancer	X						NLST [42]
Smoking history/current Tob. use							Heaviness of Smoking Index [43]
Cigarettes per day	X	X	X				
Current smoking	X	X	X				
Current use of other tobacco products	X						
Quitting history and methods used	X	X	X				
Behavioral health							K6 [44] Audit-C [45] CDE: 3600797 [46]
Depression	X	X	X				
Alcohol use	X						
Drug use	X						
Perceived risk							Risk Perception [47]
Personal and comparative risk	X	X	X				
Worry	X	X	X				
Beliefs about benefits	X	X	X				
Smoking cessation beliefs							Contemplation Ladder [48] Quitting Confidence [34] ANRT-12 [49] ISSI [50]
Readiness to quit	X	X	X				
Quitting motivation	X	X	X				
Self-efficacy	X	X	X				
Attitudes towards NRT	X						
Smoking stigma	X						
Treatment utilization and satisfaction							
NRT use		X	X				
Quitline use		X	X^b^				
Patient satisfaction		X	X^b^				
COVID-19							
Changes in motivation	X	X	X				
Changes in smoking	X	X	X				
Abstinence							
~ 3 months abstinence and 7-day prevalence		X	X				
24 h quit attempt							
Smoking reduction (50%)		X	X				
Verification of abstinence		X	X	X			
Site characteristics							
Payor mix, affiliation, screening history, % of current smokers					X	X	
Current cessation services					X	X	
Organizational priority and feasibility^c^							Organizational priority Implementation outcome Clinical Sustainability Assessment Tool [51]
Priority (readiness and barriers)					X	X	
Appropriateness and feasibility					X	X	
Sustainability						X	

^a^Not collected from site coordinators

^b^Assessed at 6 months if the 3-month survey is missed

^c^Also assessed during a semi-structured interview

### Participant surveys

Assessments developed for the study included a combination of core and opt-in items agreed upon by the members of the SCALE collaboration in addition to study-specific items [52]. Data collected from the core and opt-in items from all SCALE projects is shared with the NCI periodically and pooled to be used in cross-project collaborations.

#### Baseline variables

Our baseline assessment includes measurement of demographic characteristics (age, gender, race, ethnicity, education, marital status, education, and SES), perceived health risks (personal and comparative), worry about lung cancer, current and smoking history (frequency, number of cigarettes smoked, age of the first cigarette and current use (in the past 30 days) of other tobacco products including e-cigarettes). We also assess participants’ health literacy, self-reported physical health, medical comorbidities, family history of lung cancer, behavioral health (depression, alcohol, and illicit drug use), smoking cessation beliefs (readiness and confidence to quit, importance of quitting, and attitudes about NRT and stigma). We also added an assessment of changes in motivation and rates of smoking resulting from the COVID-19 pandemic. This collection of variables will allow us to characterize the sample and investigate moderators of treatment outcomes.

#### Process variables

SC fidelity [35, 36] to the study interventions is monitored through a review of treatment logs completed for each participant and entered via REDCap. This includes the length of time spent on each intervention, a checklist of topics discussed with the participant, and confirmation of a Quitline referral. We also collect self-reported treatment utilization data (e.g., use of NRT, receipt of interventions and services received through the Quitline) and assess satisfaction with the interventions received during the 3-month follow-up assessment [53]. Patients who do not complete the 3-month assessment are asked the satisfaction questions at 6 months.

#### Primary outcome

The primary outcome of CASTL is biochemically verified abstinence (cotinine levels reflect recent nicotine exposure) at 6 months post-randomization. Participants reporting a 7-day point prevalence abstinence at the 6-month follow-up are asked to submit a saliva sample (via mail) for analysis [54]. Consistent with intent-to-treat (ITT), unless self-reported smoking abstinence is biochemically verified, for participants who fail to return the saliva sample, the cessation outcome will be considered non-abstinent. We selected 7-day point prevalence abstinence since it is biochemically verifiable and highly correlated with continuous and sustained abstinence [55]. Salivary cotinine values of ≥ 3 ng/ml, 1 to < 3 ng/ml, and < 1 ng/ml and cotinine levels of 31.5 ng/ml, 1 to < 31.5 ng/ml, and < 1 ng/ml are consistent with active, passive, and no smoking exposure, respectively [55]. Saliva specimens are collected using mailing kits from study participants reporting smoking abstinence and sent back via overnight mail to the lab for the measurement of cotinine concentrations using an established competitive enzyme-linked immunosorbent assay (ELISA). As an alternative, we also request contact information for a proxy [56], someone who is able to confirm the participant’s abstinence in the absence of a biochemically verified sample. Participants who decline to return a saliva sample or are not confirmed abstinent by their proxy will be considered persons who smoke per ITT.

#### Secondary outcomes

In addition to the primary outcomes, secondary cessation outcomes will be self-reported 7-day point prevalence abstinence at 3 months, continuous abstinence (between the date of randomization and date of completion of follow-up surveys) at 3 and 6 months, self-reported 24 h quit attempt and reduction in cigarettes per day (cpd), changes in quitting motivation and confidence, and changes in depression and perceived smoking risk at 3 and 6 months [34, 52, 57].

#### Lung cancer screening outcomes

Results of participants’ LDCT scans are documented by each participating screening site using the standardized American College of Radiology (ACR) Lung Image Reporting and Data System (Lung-RADS) [58] of lung nodule identification, classification, and management. Lung-RADS categories range from 1 (negative), 2 (benign appearance), 3 (probably benign), and 4 (suspicious). Lung nodules categorized as category 1 and category 2 are classified as negative scans whereas category 3 and category 4 nodules are classified as positive scans. We also collect data on other incidental clinically significant or potentially significant abnormalities (i.e., S modifiers) and lung cancer diagnosis.

#### Site coordinator assessments

Data regarding SC demographics, their primary role at the lung cancer screening site, prior training as a tobacco treatment specialist, and smoking-related beliefs are collected at baseline. Baseline and follow-up assessments also include data regarding LDCT-LCS site characteristics (e.g., geographic region, patient volume, organizational priority for treating tobacco dependence, academic affiliation). We assess each site’s usual tobacco treatment practices and perceived barriers/facilitators of tobacco treatment implementation, the feasibility of implementing such processes, appropriateness (fit), and sustainability of these practices [51, 59]. These areas are also assessed during a semi-structured interview with site coordinators at the end of their participation. Interviews are recorded and transcribed for qualitative analysis.

#### PACT ancillary sub-study of SCs and Black participants

A semi-structured interview guide is used to conduct interviews with eligible Black patients who declined CASTL participation, and SCs who have screened at least 30 patients for study eligibility. The approximately 30 min interview covers topics such as barriers to tobacco treatment, prior experience with research, and reasons for tobacco treatment trial refusal [60]. The PACT semi-structured interview of SCs assesses attitudes as well as barriers, challenges, and facilitators of referring participants to the CASTL study.

#### Cost

The economic impact of the tobacco treatment components will be assessed by performing both cost and cost-effectiveness analysis. The goal of the cost analysis is to estimate the incremental costs associated with delivering tobacco treatment components in the context of LDCT-LCS. The primary endpoint of this analysis is the cost of each treatment component and the cost of each permutation of treatment components. The base-case cost analysis includes the costs of all resources consumed for the implementation and delivery of the tobacco treatment components. We will also examine costs and potential payment sources (e.g., health insurance benefits) from the provider (i.e., LDCT-LCS site) perspective, in order to estimate the net cost to screening sites that want to adopt the optimized treatment combination or individual components of it. Data sources for the cost analysis include SC treatment logs which document the delivery of treatment components and associated personnel time. Administrative and overhead costs will be estimated by each study site. The cost of medications (nicotine lozenges and patches) will be based on acquisition costs. In sensitivity analysis, we will examine a range of unit cost values for these items, reflecting the range of values reported in the medical literature and on pharmacy websites. Intervention cost estimates will include only the resources used in implementing and delivering the study interventions. Resources used solely for research purposes will be excluded.

Cost-effectiveness will be estimated as the incremental cost per additional participant with biochemically verified abstinence. This endpoint corresponds with the trial’s primary clinical endpoint and will facilitate comparison with other smoking cessation trials that estimate cost-effectiveness as cost per quit [61]. In addition to intervention costs, the numerator of the cost-effectiveness ratio will include the costs of smoking cessation supplies consumed after the study treatment period and other non-study tobacco treatment services received and the patient time and travel costs associated with these services. The use of additional tobacco treatments and time and travel costs will be reported by participants in the 3- and 6-month follow-up surveys. Incremental cost-effectiveness ratios will be estimated for all non-dominated intervention strategies, and uncertain parameters will be varied in sensitivity analysis across a range of plausible values.

### Data analysis

#### Analytic strategies to address research aims

*Aim 1: To identify which of four evidence-based tobacco treatment components contribute to superior cessation endpoints among persons who currently smoke seeking lung cancer screening over and above enhanced standard care.*

In addition to descriptive statistics, a generalized hierarchical linear mixed effects modeling approach will be used to address the primary effectiveness aim. This approach will account for the nested structure of the data (i.e., patients nested within sites) via random intercepts and allow the use of a logit link function for the binary outcome of abstinence. With the factorial design of this study, a fully saturated model includes all four main component effects, 6 two-way interactions, 4 three-way interactions, and 1 four-way interaction.

The main analytic strategy involves a model simplification process. The saturated model will be fitted first. Next, model terms with a statistically reliable effect (by *p* < 0.05 in the type 3 analysis of variance table) will be retained. We envisage that some synergistic effects (interactions) will emerge and be deemed the optimized tobacco treatment package. All analyses will be guided by the intention-to-treat principle [62], in which participants with missing outcomes will be deemed non-abstinent, and thus, missing tobacco abstinence outcomes will not be imputed. To control for false discovery, the selection of model terms will generally be guided by the adjusted *p*-values by using the method of Benjamini and Yekateuli (2001) [63] for dependent false discovery rate because there may exist plausible correlations in the *p*-values between the main effects and the interaction terms.

#### Statistical power and sample size considerations for the primary aim

Using the statistical power calculation computer programs designed specifically for MOST studies [64] and accounting for up to 20% attrition, the anticipated sample will afford an 85% statistical power (type I error at two-sided 5%), at an estimated minimal effect size of 0.20 in standard deviation units, what Cohen would consider a “small” effect in behavioral research [65]. The 0.20 minimal effect size represents the smallest main effect that can be supported by the proposed sample size in the MOST intervention components. For illustration, a difference between 20 and 12% abstinence rates, when converted by an arcsine transformation, corresponds to a Cohen’s *d* = 0.22.

*Aim 2. To estimate the cost and incremental cost-effectiveness of evidence-based tobacco treatment components, delivered alone and in combination.*

The base-case cost analysis will take a societal perspective, estimating all costs associated with treatment components delivered separately and in combination. We will conduct a separate cost analysis from the provider’s perspective. We will use standard methods [66] of incremental cost-effectiveness analysis to estimate the additional cost per quit achieved, where cost includes both intervention costs and 6-month downstream costs of related health care tobacco cessation services, and the effectiveness measure is defined by 7-day point prevalence abstinence at 6 months.

Given the primary focus of the trial on non-economic endpoints, and sample size requirements associated with these endpoints, formal hypothesis testing on the economic outcomes will not be conducted. Resource utilization and cost data are typically skewed, and therefore, the sample size of the trial may be insufficient to detect significant differences in costs between study arms [67]. The economic impact of the intervention will be evaluated using standard incremental cost-effectiveness analysis methods. For example, it is possible that several treatment combinations may yield comparable effectiveness in abstinence. If this happens, then the incremental cost per abstinence may be factored into this consideration. Sensitivity analysis will be conducted to assess the impact of assumptions and uncertainty on results and conclusions [66, 68]. This analytic approach is appropriate in economic studies that “piggyback” randomized trials [69].

*Aim 3. To conduct a robust, mixed methods evaluation of the implementation process and assess site characteristics and organizational factors that may influence the implementation of tobacco treatment in lung cancer screening settings.*

Descriptive statistics will be used to summarize key implementation outcomes (reach, adoption, fidelity, acceptability, appropriateness) collected from SC surveys [25]. Site characteristics will also be used in exploratory analyses on variation in cessation and implementation outcomes to examine the extent to which site characteristics are associated with intervention effectiveness, fidelity, and acceptability. To better understand the implementation challenges and sustainability, semi-structured interviews with SCs will be conducted following the completion of study participation. Interviews will be audio-recorded and transcribed. Led by an experienced qualitative methods specialist (QMS), a team of trained and supervised coders will analyze the transcripts using NVivo Pro version 12.0, a qualitative data analysis management software program [70]. Coders will utilize a thematic text analysis approach, which will occur in two phases [60, 71, 72]. In the first phase, the team will iteratively develop a codebook based on a priori domains and inductive concepts that emerge from the data, meeting regularly to reach a consensus on code name and definition. Then, all transcripts will be independently coded, and the QMS will complete a quality assurance check of the dataset. In the second phase, the team will group coded statements into analytic domains, and review these categories to identify and describe major and minor thematic areas. Themes will include sentiments that appear across most transcripts, as well as any significant divergences.

*Aim 4: To analyze whether lung cancer screening results (i.e., Lung-RADS) moderate smoking abstinence outcomes.*

We will use the Lung-RADS category score (1–4) as an ordinal measure of lung cancer screening findings and examine whether the proportion of participants achieving smoking abstinence differs by the degree of screening abnormalities (Lung-RADS score).

For the PACT ancillary study, interview transcripts with the SCs and PACT participants will be coded utilizing a grounded theory approach, consisting of open, axial, and selective coding phases [73]. Analytic software NVivo Pro v. 12.0 will be used to facilitate the analysis [70]. Using the grounded theory approach, two coders will independently code each transcript with the goal of identifying and describing themes that persistently emerge as barriers to recruitment to CASTL, meeting regularly to reach a consensus on code names, definition, and assignment to content. Each phase of analysis will be used to iteratively generate a theory to inform reasons for tobacco treatment trial refusal among Black participants.

### Data management and monitoring

A multidisciplinary study team was assembled to lead and manage the day-to-day responsibilities of this study. This team includes MPIs from MSK—the study sponsor and data coordinating center—and New York University, study staff based at MSK including co-investigators, a project manager, CRCs, and regulatory support staff who will be responsible for the day-to-day management of the study and research staff at the GO2 will work in partnership with the CASTL study team to identify and recruit participating screening sites. The core study team—the MPIs, MSK study staff, and GO2 staff—will meet bi-weekly to oversee study conduct and overall progress. Additional team members will include consultants from multiple institutions^1^ around the country and screening site staff—a site PI and site coordinator. Site coordinators will be responsible for referring potential participants to the study and providing study interventions. Bi-weekly meetings will be conducted with site coordinators, the MPIs, MSK study team, and GO2 representatives for supervision intended to promote adherence to the study protocol and provide ongoing support to the SCs.

The survey data collected is managed via the study’s REDCap database by the RPA, CRC(s), and Clinical Research Supervisor [74]. To ensure confidentiality of data, all records, including hard copies of study documents, will be identified using the participant’s unique study identifier, not by name, and will be stored in a locked secure area at MSK. Only the PI and MSK research staff will have access to the study files and REDCap records. Recorded site coordinator interviews will be uploaded to a secured shared drive at MSK. Biospecimens (saliva samples) collected for the bio-verification of abstinence will be assigned a unique identifier and stored at an MSK laboratory. Any portion of the sample remaining after testing is completed will be discarded and will not be stored for use in future ancillary studies. Although this is a minimal risk trial, it is monitored by the MSK Data Safety and Monitoring Board (DSMB), which is composed of a plurality of voting members who are not affiliated with MSK. Random-sample data quality and protocol compliance audits will be conducted by the study team, at a minimum of two times per year, more frequently if indicated.

Protocol amendments will be managed by the study team at MSK, the IRB of record. Participating sites will be notified of amendments and copies of revised protocol will be shared with sites for addition to their regulatory binders. Any deviations from the protocol, adverse events (AEs), and/or serious adverse events (SAEs) will be reported to the PIs and submitted to MSK’s IRB for review.

### Criteria for removal and adverse events

We do not anticipate any serious adverse events that are detrimental to study participants to occur as this is a minimal-risk trial. In the unlikely event that a participant expresses distress, the research staff will refer to appropriate assistance at each site as needed, or to the study PIs if appropriate. It will be made clear to all study participants (SCs and smokers) that they are allowed to withdraw from the study at any time or discontinue the use of the medication if they experience serious adverse reactions. Participants will be asked if they would like their data to be excluded from the trial at the time of withdrawal as long as that data has not already been otherwise used or shared. These requests must be received in writing. Participants are informed that there is no anticipated harm or compensation for post-trial participation beyond incentives for survey completion. If at any time the participant is found to be ineligible for the protocol (e.g., they are ineligible for lung cancer screening or a contraindication with combination NRT is discovered post-randomization) as designated in the section on criteria for patient/subject eligibility, the participant will be removed from the study. We will also remove patients who do not complete the baseline assessment from the study. Allocated interventions will not be otherwise discontinued or modified.

### Data sharing and dissemination plan

Participants will be asked for permission for the research team to share relevant data with study collaborators and regulatory bodies such as the NCI. Requests made by other researchers for the study protocol and/or data will be considered by the study sponsor. For annual data transfers to the NCI, de-identified data will be shared through a secure online portal managed by the NCI in accordance with the data transfer agreement for analysis and use in related collaborative projects. The datasets analyzed during the current study and statistical code are available from the corresponding author on reasonable request, as is the full protocol. Any shared data will be de-identified.

Study findings will be disseminated primarily through peer-reviewed manuscripts and presentations at relevant scientific conferences. Findings will also be disseminated by collaborating colleagues from the National Cancer Institute, the GO2 Foundation, and the National Lung Cancer Roundtable.

## Discussion

Lung cancer screening provides a unique opportunity to engage current tobacco users in cessation efforts. Integration of effective evidence-based tobacco use treatment methods may increase the benefits of LDCT-LCS, ultimately improving health outcomes in an older population of people who smoke. This paper describes the CASTL clinical trial using the MOST factorial design to test the effectiveness of several tobacco treatment interventions under investigation including referrals to the national Quitline, nicotine replacement therapy (patch and lozenge), motivational counseling, and message framing delivered to patients seeking lung cancer screening at LDCT-LCS sites nationwide.

Using a hybrid type II trial design [75], we will generate data to support the selection of an optimal combination of tobacco use treatment interventions for patients undergoing LCS while explicitly collecting data on implementation processes (e.g., patient and provider satisfaction, fidelity, cost) to facilitate subsequent dissemination and implementation efforts. The main deliverable in this trial will be the optimal intervention components. More specifically, if, hypothetically speaking, intervention combinations 3, 4, 7, and 8 in Table 1 yield the highest abstinence rates, then the optimal intervention would involve a synergic effect between motivational interviewing and nicotine patch in addition to enhanced standard care. This determination will have to be made empirically, after the trial has concluded and data analyzed.

Our comprehensive evaluation of both effectiveness and contextual aspects of implementation across a diverse group of LDCT-LCS settings will inform LDCT-LCS program decisions about necessary organizational structures, resources, and processes needed to integrate tobacco dependence treatment. Given the diversity of current cessation practices in the LDCT-LCS setting, this comprehensive approach will allow for the most robust data to inform implementation practices across different program sizes, geographic regions, academic affiliations, and other LDCT-LCS program characteristics.

### Limitations

Careful consideration was given to potential pitfalls and methodological challenges that may be encountered during the proposed project. First, we recognize the concern about potential treatment contamination and have taken several steps to ensure that participants receive the treatment package consistent with their random assignment. Random assignment of patients to treatment conditions is conducted centrally by the MSK Coordinating Center and the appropriate treatment components are mailed to participating patients and their SC is informed of the assignment by email. We will also assess implementation fidelity with several sources of triangulated data. Third, given that CMS required that persons who currently smoke receive information about treatment for tobacco dependence, we decided to evaluate potential tobacco treatment component enhancements against a high quality, enhanced standard of care rather than a no treatment control. Finally, we considered several alternate treatment components and eliminated tobacco treatment options unlikely to be feasible (e.g., prescription medications) or sustainable (intensive group or individual counseling) in most LDCT-LCS settings. It is plausible that more intensive tobacco treatment components may be needed to achieve clinically meaningful cessation outcomes and long-term smoking abstinence.

### Clinical implications

The CASTL trial findings will provide a national model for best practices of tobacco treatment delivery in LDCT-LCS settings. Our findings will also establish a strong empirical foundation for testing implementation strategies [76] for wider dissemination of effective treatment models in LDCT-LCS settings based on our comprehensive evaluation of implementation outcomes (e.g., fidelity, cost, acceptability, appropriateness, barriers to sustainability), findings of engagement (i.e., who enrolled and participated) and measures of tobacco treatment utilization (reasons for nonadherence), patient satisfaction with treatment, and treatment effectiveness. By pooling data and conducting cross-projects analyses in collaboration with the other NCI-funded SCALE studies, this work will greatly contribute to generalizable knowledge regarding the integration and dissemination of evidence-based tobacco cessation into a wide range of LDCT-LCS settings.

In summary, the CASTL trial has multiple strengths, including a robust partnership with academic and community-affiliated lung cancer screening sites across the USA, broad patient inclusion criteria that will allow results to be generalizable to most persons who some seeking LDCT-LCS, biochemical verification of smoking abstinence and mixed methods examination of implementation processes essential for scaling up the adoption of evidence-based tobacco treatment in LDCT-LCS settings.

## Trial status and modifications

Protocol version: A(13) Approved 03/03/2022.

Patient enrollment began in August 2018 and is ongoing (currently enrolled: *n* = 734). We anticipate completing recruitment in Fall 2022 and data collection in Spring 2023, after which analysis will begin. The COVID-19 pandemic had a marked impact on participant recruitment resulting from site closures and redeployment of screening site staff. The research team has made several modifications designed to expand recruitment timelines in order to help reduce staff burden and facilitate study implementation across the sites. These included (1) increasing recruitment timelines from 7 business days post LDCT scan to a period of 120 business days (60 pre/60 post) LDCT scan to allow site coordinators to look forward or backward on their schedules, (2) allowing the study PIs and staff to deliver interventions if needed, (3) reducing the age and smoking history requirements to match the updated USPSTF guidelines for screening, and (4) providing additional incentive for the timely return of saliva samples. All of these changes were approved by the IRB prior to implementation. Due to the continued disruptive impact of COVID-19, we have opted to not open our 18th screening site to recruitment. We have also decided to close some non-performing sites. As a result of these pragmatic decisions, we will not be able to achieve the initially planned 1152 participants for this study.

## Data Availability

The study PIs and MSK study team will have access to the final study dataset. External study collaborators will have access to de-identified data.

## Abbreviations

MSK: Memorial Sloan Kettering Cancer Center
NCI: National Cancer Institute
LDCT: Low-dose computed tomography
LDCT-LCS: Low-dose computed tomography lung cancer screening
NRT: Nicotine replacement therapy
PRO-CEL: Patient Reported Outcomes and Community Engagement and Language Core
MOST: Multiphase Optimization Strategy
SCALE: Smoking Cessation at Lung Examination
CASTL: Cessation and Screening to Save Lives
SC(s): Site coordinator(s)
CRC(s): Clinical research coordinator(s)
PACT: Participation of African-Americans in Cessation Trials
REDCap: Research Electronic Data Capture
MI: Motivational interviewing
SCOE: Screening Centers of Excellence
DSMB: Data Safety and Monitoring Board

## Footnotes

1 Columbia University, Rutgers University, University of Colorado and the University of Washington
